# Metagenome-based diversity analyses suggest a significant contribution of non-cyanobacterial lineages to carbonate precipitation in modern microbialites

**DOI:** 10.3389/fmicb.2015.00797

**Published:** 2015-08-05

**Authors:** Aurélien Saghaï, Yvan Zivanovic, Nina Zeyen, David Moreira, Karim Benzerara, Philippe Deschamps, Paola Bertolino, Marie Ragon, Rosaluz Tavera, Ana I. López-Archilla, Purificación López-García

**Affiliations:** ^1^Unité d’Ecologie, Systématique et Evolution, CNRS UMR 8079, Université Paris-SudOrsay, France; ^2^Institut de Génétique et Microbiologie, CNRS UMR 8621, Université Paris-SudOrsay, France; ^3^Institut de Minéralogie et de Physique des Matériaux et de Cosmochimie, CNRS UMR 7590, Université Pierre et Marie CurieParis, France; ^4^Departamento de Ecología y Recursos Naturales, Universidad Nacional Autónoma de MéxicoMexico City, Mexico; ^5^Departamento de Ecología, Universidad Autónoma de MadridMadrid, Spain

**Keywords:** stromatolite, biomineralization, metagenomics, cyanobacteria, anoxygenic photosynthesis, sulfate-reduction, diatoms, green algae

## Abstract

Cyanobacteria are thought to play a key role in carbonate formation due to their metabolic activity, but other organisms carrying out oxygenic photosynthesis (photosynthetic eukaryotes) or other metabolisms (e.g., anoxygenic photosynthesis, sulfate reduction), may also contribute to carbonate formation. To obtain more quantitative information than that provided by more classical PCR-dependent methods, we studied the microbial diversity of microbialites from the Alchichica crater lake (Mexico) by mining for 16S/18S rRNA genes in metagenomes obtained by direct sequencing of environmental DNA. We studied samples collected at the Western (AL-W) and Northern (AL-N) shores of the lake and, at the latter site, along a depth gradient (1, 5, 10, and 15 m depth). The associated microbial communities were mainly composed of bacteria, most of which seemed heterotrophic, whereas archaea were negligible. Eukaryotes composed a relatively minor fraction dominated by photosynthetic lineages, diatoms in AL-W, influenced by Si-rich seepage waters, and green algae in AL-N samples. Members of the Gammaproteobacteria and Alphaproteobacteria classes of Proteobacteria, Cyanobacteria, and Bacteroidetes were the most abundant bacterial taxa, followed by Planctomycetes, Deltaproteobacteria (Proteobacteria), Verrucomicrobia, Actinobacteria, Firmicutes, and Chloroflexi. Community composition varied among sites and with depth. Although cyanobacteria were the most important bacterial group contributing to the carbonate precipitation potential, photosynthetic eukaryotes, anoxygenic photosynthesizers and sulfate reducers were also very abundant. Cyanobacteria affiliated to Pleurocapsales largely increased with depth. Scanning electron microscopy (SEM) observations showed considerable areas of aragonite-encrusted *Pleurocapsa*-like cyanobacteria at microscale. Multivariate statistical analyses showed a strong positive correlation of Pleurocapsales and Chroococcales with aragonite formation at macroscale, and suggest a potential causal link. Despite the previous identification of intracellularly calcifying cyanobacteria in Alchichica microbialites, most carbonate precipitation seems extracellular in this system.

## Introduction

Calcifying microbial mat communities may result in the formation of microbialites (Riding, 2000; Tice and Lowe, 2004; Dupraz and Visscher, 2005). These can be laminated (stromatolites) or not (thrombolites). Because they are lithified, microbialite communities can be more easily preserved in the fossil record than non-calcifying microbial mats and, when they are laminated at macroscale, they provide a simple recognizable morphological diagnosis for biogenicity. It is indeed this kind of morphology that makes fossil stromatolites from the early Archaean (3.43 Ga old) the oldest and less disputed life traces on Earth (Awramik, 1990; Altermann, 2004; Tice and Lowe, 2004; Allwood et al., 2006, 2009). Although conspicuous and widely distributed throughout the Proterozoic (2.5 Ga – 0.5 Ga), stromatolites declined afterward (Awramik, 1971; Altermann et al., 2006). Today, microbialites are restricted to a few marine sites, notably the hypersaline Shark Bay in Western Australia (Logan, 1961; Awramik and Riding, 1988) and the nearly open marine environment of Highbourne Cay in the Bahamas (Dravis, 1983; Reid and Browne, 1991), and to a variety of inland water bodies. These include some hypersaline systems, such as high-altitude Andean hypersaline ponds (Farias et al., 2013, 2014), but also freshwater systems in karst areas, such as the Pavilion Lake (Laval et al., 2000), Cuatro Ciénegas wetlands (Breitbart et al., 2009), or Ruidera Pools (Santos et al., 2010), and in volcanic areas, such as the Lake Van (Kempe et al., 1991; Lopez-Garcia et al., 2005) or the crater Lake Alchichica (Kazmierczak et al., 2011).

In modern systems, two major processes can lead to microbialite formation, the accretion of small particles and the *in situ* precipitation of minerals, mostly carbonates, due to microbial activity. Whereas the accretion of particles, essentially by filamentous cyanobacteria (Reid et al., 2000) and/or algae (Awramik and Riding, 1988), plays a major role in marine systems such as the Bahamas or Shark Bay, microbial-induced precipitation seems to dominate in freshwater systems, such as crater lake microbialites. This makes them more similar to some Precambrian stromatolites, such as those of the massive 2.7 Ga-old Tumbiana formation (Bolhar and Van Kranendonk, 2007), and potentially others (Kazmierczak and Kempe, 2006; Kazmierczak et al., 2011). Understanding how the activity of microbial communities in those settings results in mineral precipitation may therefore provide insights on how the first microbialites formed. For carbonate precipitation to occur, three requisites must concur: excess carbonate ions (super-saturation), the availability of free cations (e.g., Ca^2+^ or Mg^2+^) and the presence of nucleation centers (Dupraz and Visscher, 2005). It is generally believed that the abundant exopolymeric substances (EPS) produced by many cyanobacteria are a source of cations and nucleation centers (Benzerara et al., 2006; Dupraz et al., 2009; Obst et al., 2009). Also, microbial activity can lead to carbonate super-saturation by increasing the local pH and deviating the CO_2_ dissolution equilibrium toward CO_3_^2-^. Several metabolisms are known to promote carbonate super-saturation, including oxygenic and anoxygenic photosynthesis (Dupraz and Visscher, 2005; Bundeleva et al., 2012), but also sulfate reduction (Visscher et al., 2000; Gallagher et al., 2012), or anaerobic methane oxidation coupled to sulfate reduction (Michaelis et al., 2002). Others, on the contrary, tend to promote dissolution by acidification, such as aerobic respiration, sulfide oxidation, or fermentation (Dupraz and Visscher, 2005). The net production of carbonates will finally depend on the balance activity of microorganisms bearing those different metabolisms.

In the past few decades, it has become clear that microbialite microbial communities are extremely diverse and that members from very different taxa can display similar metabolisms sustaining an ‘alkalinity engine.’ Thus, although for a long time stromatolites were thought to be formed by the photosynthetic activity of cyanobacteria (‘blue–green algae’), the discovery of anoxygenic photosynthesis in Chloroflexi, also present in calcifying mats and stromatolites, revealed the importance of other photosynthesizers in these systems (Doemel and Brock, 1974). Today, we know that other bacterial taxa, including Chlorobi, Alphaproteobacteria and Gammaproteobacteria, which are frequent members of microbialite communities, are able to carry out anoxygenic photosynthesis (Madigan et al., 2014). In addition, molecular analyses of prokaryotic diversity based on amplification of 16S rRNA genes followed by classical cloning and Sanger sequencing or by more recent high-throughput amplicon sequencing techniques have revealed an extraordinary diversity of bacteria in microbialites from both marine (Burns et al., 2004; Papineau et al., 2005; Allen et al., 2009; Foster et al., 2009; Goh et al., 2009; Myshrall et al., 2010) and freshwater settings (Lopez-Garcia et al., 2005; Couradeau et al., 2011). Among the many bacterial phyla and candidate divisions identified, Proteobacteria (specifically members of the Alphaproteobacteria and Gammaproteobacteria classes), Cyanobacteria, Bacteroidetes, and Planctomycetes seem to be among the most abundant and diverse, whereas Archaea are scarce and with very low diversity. Only a few more recent studies have also focused on the analysis of eukaryotic diversity based on 18S rRNA gene amplicon sequencing, including marine stromatolites at Highbourne Cay (Myshrall et al., 2010) and Shark Bay (Allen et al., 2009), high altitude non-lithified calcifying mats in Atacama (Farias et al., 2013) and the freshwater crater lake Alchichica (Couradeau et al., 2011). This seemingly lesser interest in microbialite-associated eukaryotes may be related to the fact that the oldest Precambrian stromatolites could only derive from prokaryotic activities before eukaryotes evolved (Allwood et al., 2006) and to the general idea that cyanobacteria are the major (if not the exclusive) biological contributors to microbialite formation (Reid et al., 2000; Foster et al., 2009). However, eukaryotes may have well contributed to ancient microbialite formation since they appeared around 2 Ga ago (Javaux, 2007; Parfrey et al., 2011). Indeed, the diversity of microbialite eukaryotes is larger than currently thought, including many chlorophytes, ciliates, foraminifera, diatoms, and other stramenopiles, fungi, amoeba, and several deep-branching lineages (Couradeau et al., 2011; Edgcomb and Bernhard, 2013), and many of these eukaryotes may have cohesive and/or calcifying functions. For instance, stromatolite-associated filamentous algae can trap grains (Awramik and Riding, 1988), foraminifera can stabilize particles (Bernhard et al., 2013) and eukaryotic algae may promote carbonate precipitation via their photosynthetic activity (Riding, 2000).

However, even if recent molecular analyses based on amplicon sequencing provide qualitative information about the diversity of archaea, bacteria and eukaryotes, the relative inter-domain proportions remain essentially unknown because primer pairs used to amplify 18S and 16S rRNA genes are generally domain-specific. Moreover, in addition to common biases that affect all kind of molecular analyses, such as differential cell lysis, DNA purification yield or gene copy number, classical 16S/18S rDNA amplification and cloning strategies are affected by both PCR- (Acinas et al., 2005) and cloning-related (Temperton et al., 2009) biases. More recent high-throughput sequencing technologies yielding millions of relatively shorter sequences, although unsuitable for deep phylogenetic affiliation, may be more relevant to infer the actual structure of complex microbial communities (Caporaso et al., 2011). However, they still suffer from PCR-related biases (Haas et al., 2011; Pinto and Raskin, 2012). In this sense, mining for 16S/18S rDNAs identified in datasets generated by direct metagenomic sequencing (e.g., via Illumina) of bulk DNA may be more accurate at retrieving the actual composition of microbial communities than amplicon-based strategies (Shah et al., 2011; Shakya et al., 2013; Logares et al., 2014). This approach, while having its own limitations (e.g., variation in rDNA genes copy number may affect semi-quantitative analyses), is free from both PCR and cloning-related biases (Logares et al., 2014). So far, microbialite metagenomic datasets have been generated for Cuatro Ciénegas (Breitbart et al., 2009) and for calcifying and non-calcifying mats from Bahamian stromatolites (Khodadad and Foster, 2012) by 454 pyrosequencing. While these studies provided interesting insights into the microbial diversity and functional potential of those communities, the number of reads generated by 454 pyrosequencing is far lower than that of more recent techniques (e.g., Illumina), which severely limits the total number of rDNA sequences that can be identified especially for lower abundance taxa. In addition, due to the inherent difficulties to purify DNA from microbialite samples (Wade and Garcia-Pichel, 2003), genome amplification by multiple displacement was applied to DNA samples from Cuatro Ciénegas (Breitbart et al., 2009), making quantitative estimates problematic.

In this study, we have analyzed the diversity of 16S and 18S rRNA genes in metagenomes generated by direct Illumina sequencing from several microbialite samples collected at different sites and depths in Lake Alchichica in parallel with their mineral composition. Alchichica is an alkaline (pH ∼9) crater lake located at high altitude (2,300 m above sea level), which harbors conspicuous microbialites consisting essentially of hydromagnesite [Mg_5_(CO_3_)_4_(OH)_2_.4(H_2_O)] and aragonite (CaCO_3_), (Kazmierczak et al., 2011; Couradeau et al., 2013). Prior molecular diversity analyses based on 16S and 18S rDNA amplicon cloning and sequencing revealed a broad diversity of bacteria and eukaryotes, with a minor presence of archaea (Couradeau et al., 2011). Cyanobacteria from the order Pleurocapsales appeared to be relatively abundant in deeper microbialites and to become specifically encrusted in aragonite at microscale (Couradeau et al., 2013; Gerard et al., 2013). The objectives of our study were to (i) characterize the diversity of microorganisms from the three domains of life in a more quantitative way and with higher sequence coverage, (ii) gain insight into the relative abundance of taxa potentially involved in carbonate precipitation and, (iii) correlate the observed diversity with local environmental parameters such as depth and mineral composition. This should help testing specific relationships among taxa and particular mineral occurrence at macroscale.

## Materials and Methods

### Sample Collection and Processing

**FIGURE 1:**
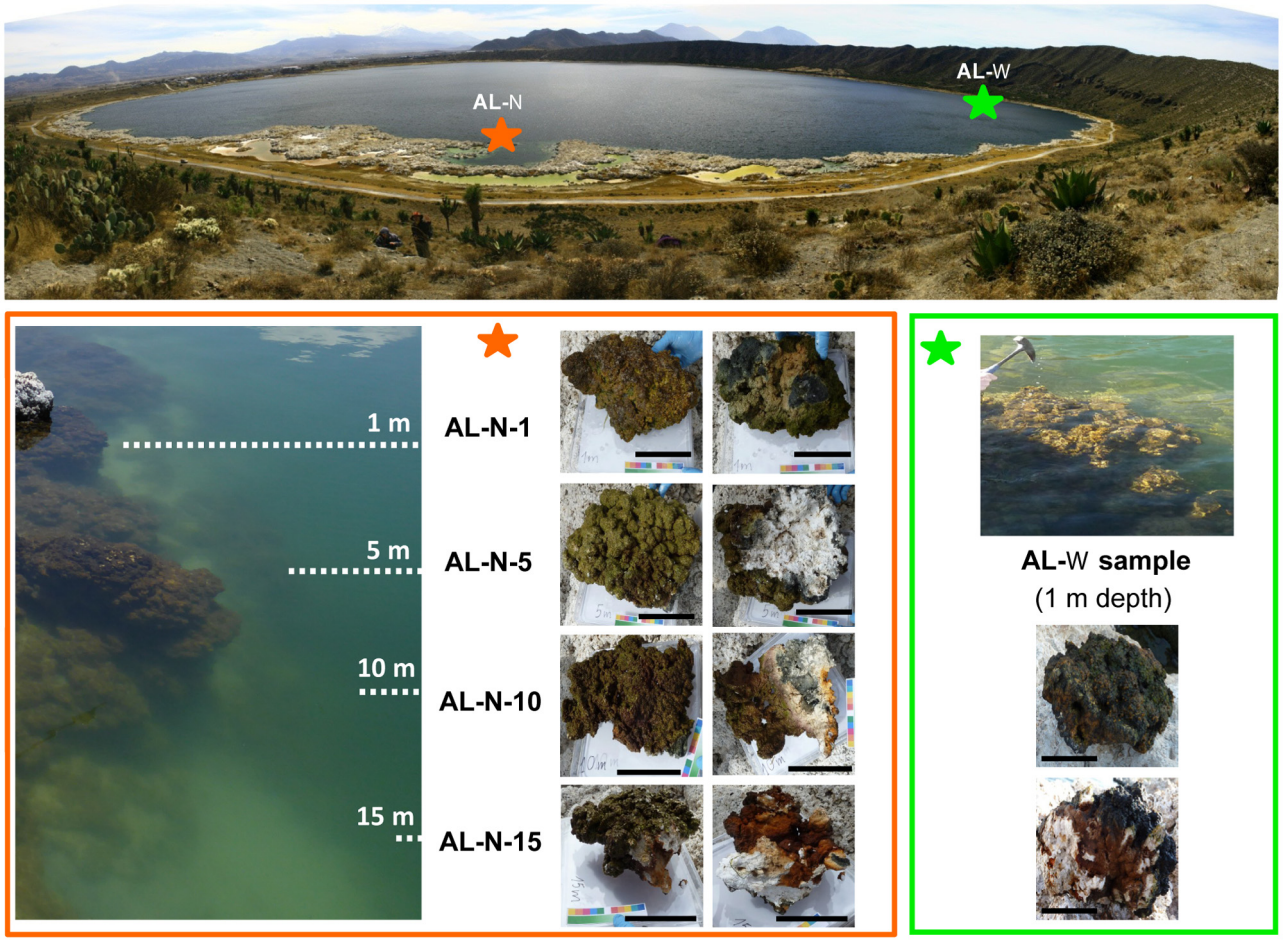
**Lake Alchichica sampling sites and microbialite fragments collected.** The two sampling sites AL-N and AL-W are indicated on the lake with stars. Photographs taken on site of the different samples are shown below. For AL-N samples, the correspondence with the depth gradient is schematically indicated on the **(left)**. Upper views of microbialite fragments are shown on the **left** (AL-N) and **upper** (AL-W) part of the respective panels; downside views are on the **right** (AL-N) and **lower** (AL-W) part of the panels. The scale bar corresponds to 10 cm.

Microbialite samples were collected in January 2012 during the winter season when the lake is not stratified (Macek et al., 2009). Samples were collected in two different sites of the lake: the Western shore (19° 25′ 0.13″N; 97° 24′ 41.07″W) and the Northern shore (19° 25′ 12.49″N; 97° 24′ 12.35″W), which we termed AL-W and AL-N, respectively. The AL-W site was located at the steepest shore of the lake, where seepage activity was visible (active bubbling observed at the shore); the AL-W sample was collected at ca. 0.5–1 m depth with the help of a geologist’s hammer. At the AL-N site, samples were collected at different depths (1, 5, 10, and 15 m depth) by scuba diving. A portion of each microbialite fragment was removed and let dry for parallel chemical and mineralogical analysis. For metagenomic analysis, fragments of ca. 20 cm in diameter were picked up with gloves, photographed on site (**Figure 1**) and subsequently broken and manipulated with sterile chisels and/or forceps to minimize all possible contamination. For DNA purification, millimeter-sized microbialite grains were detached and collected from the total surface of those microbialite fragments down to a few millimeters deep in order to get predominantly the active microbialite community and to facilitate subsequent grinding prior to DNA purification. A large surface was sampled to limit local heterogeneity effects. AL-W samples were covered by a more glutinous and less mineralized biofilm, whereas AL-N samples were mineralized up to the surface (**Figure 1**). Subsampled grains were placed in sterile 50-ml Falcon tubes and transported to the UNAM laboratory (244 km away) at 4°C for immediate DNA purification. The remainder of the samples were frozen and stored at –20°C for subsequent use.

### Analysis of Chemical and Mineralogical Composition

**FIGURE 2:**
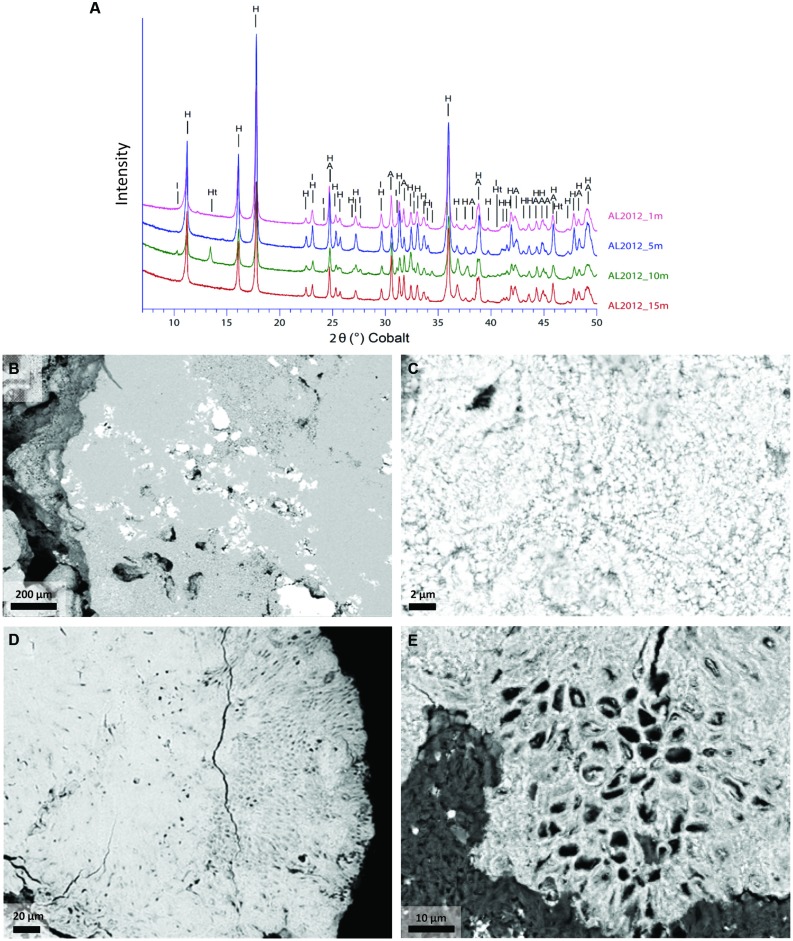
**Mineral phases of Alchichica microbialites. (A)** X-ray diffractograms of the Alchichica microbialite fragments sampled at different depths on the Northern shore (AL-N samples). **(B–E)** Scanning electron microscopy (SEM) images of the sample AL-N-10 (10 m depth) showing the most conspicuous mineral phases; hydromagnesite (H), aragonite (A), hydrotalcite (Ht), and illite (I) can be identified from the XRD patterns. **(B)** Hydromagnesite (light gray areas occupying most of the scanned surface) and aragonite (bright areas). **(C)** Micritic aragonite. **(D,E)** Large areas showing the encrustation of *Pleurocapsa*-like cyanobacteria in aragonite (the organic matter present in living cells appears in dark gray).

Concentrations of major elements, total organic carbon, and total sulfur were determined at the *Service d’analyse des roches et minéraux* (SARM), Centre de Recherches Pétrographiques et Géochimiques, Nancy, France (**Table 1**). Approximately 2 g of dry ground powder per sample were used for the analyses. Major elements were quantified using an ICP-AES ICap 6500 (Thermo Fischer) after an alkali fusion of rock powder with LiBO_2_ followed by dissolution with HNO_3_. The uncertainties of the major element measurements were between 1 and 25% depending on their concentrations. They were better than 2, 2, and 5% for Ca, Mg, and Si, respectively, which were considered for calculating the mineralogical composition of the microbialites. Total sulfur content was determined using the carbon/sulfur analyzer Horiba EMIA320V2. The uncertainties of these measurements were better than 15% for sulfur concentration values. The bulk mineralogical composition of all the samples was first analyzed by x-ray diffraction (XRD). About 1 g of each microbialite sample was crushed in an agate mortar and the powder was deposited on an aluminum sample holder. XRD measurements were performed using a PANalytical X’Pert diffractometer equipped with a cobalt anode (Co-Kα). Data were recorded at 40 kV and 35 mA in the continuous-scan mode between 4 and 120° (2θ) with a step of 0.0167° and a total counting time of around 2 h. XRD data were analyzed using the PANalytical X’Pert Highscore software for background subtraction, peak finding, and matching with XRD patterns of reference compounds from the International Crystal Structure Database (ICSD, Fachinformationszentrum, Karlsruhe, Germany; US Institute of Standards and Technology, USA). All samples were composed of two major carbonate phases: hydromagnesite [Mg_5_(CO_3_)_4_(OH)_2_.4H_2_O] and aragonite (CaCO_3_). In addition, we were able to identify hydrotalcite [(Mg _0.667_ Al_0.333_)(OH)_2_ (CO_3_)_0.167_ (H_2_O)_0.5_] and illite (K_4_Al_16_Si_8_O_48_) as minor phases in some samples (**Figure 2**). Moreover, based on scanning electron microscopy (SEM) analyses (Zeyen et al., under review) and the present chemical analyses, we considered an additional silicate phase with a talc-like composition: Mg_3_Si_4_O_10_(OH)_2_. Based on this mineralogical assemblage and bulk chemical analyses of the samples, we could calculate the proportion of these three different phases. SEM observations of ultrathin sections coated with carbon were done in angle selected backscattered electron mode using a Zeiss Ultra 55 FEG-SEM operating at 15 kV at a working distance of 7.5 mm.

**Table 1:** Proportion of major elements and minerals present in Alchichica microbialite samples.

Sample name	AL-W	AL-N-1	AL-N-5	AL-N-10	AL-N-15
**Elemental composition (% of the total mass)**					
Total S	0.12	0.15	0.05	0.08	0.13
Si	0.56	1.15	1.61	8.20	0.96
Al	0.12	0.27	0.21	1.90	0.17
Fe	0.28	0.13	0.07	0.25	0.14
Mn	0.0085	0.0089	0.0051	0.02	0.01
Mg	31.16	31.57	34.86	28.56	24.2
Ca	10.54	10.45	8.18	11.94	20.9
Na	0.28	0.31	0.20	0.65	0.43
K	0.04	0.06	0.04	0.25	0.05
P	0.04	0.04	0.05	0.04	<L.D.
**Mineral composition (% of the total mass)**					
Aragonite	24	23	17	16	55
Hydromagnesite	75	75	80	58	44
Talc	1	2	3	16	1

### DNA Purification and Sequencing

DNA extraction took place immediately upon return. Microbialite grains were ground using a sterile agate mortar and the material that was not immediately used for DNA purification stored at –20°C. DNA purification from individual microbialite powder aliquots of 0.2 g was carried out using the Power Biofilm^TM^ DNA Isolation Kit (MoBio, Carlsbad, CA, USA) according to manufacturer’s instructions. To facilitate the lysis of cells within the carbonate matrix and limit DNA adsorption to carbonate, which seriously diminish DNA yield (Wade and Garcia-Pichel, 2003), we previously applied a quick acid treatment followed by immediate neutralization as described (Couradeau et al., 2011). Briefly, 100 μl of 33% HCl were added to each 0.2-g powder aliquot for 30 s, then immediately neutralized with 1 ml of a 1:1 mixture of PBS and 0.5 M EDTA, pH 9. The total genomic DNA sent to sequence was pooled from several DNA purification products to minimize potential biases due to potential sample and process heterogeneity. DNA recovered from 20 powder aliquots (a total of 4 g) was pooled for AL-W, with a final yield of 22 μg. The yield of AL-N samples was much lower, reflecting the more imbricated nature of microbial biofilms within the carbonates. Pooling the DNA extracted from 32 independent powder aliquots per sample (a total of 6.4 g per sample), the amounts of genomic DNA recovered were 1.4 μg (AL-N-1), 0.9 μg (AL-N-5), 0.8 μg (AL-N-10), and 0.85 μg (AL-N-15). Pooled DNAs recovered from AL-N extractions were further cleaned using the Power Clean^TM^ Pro DNA Clean-up Kit (Mo Bio, Carlsbad, CA, USA), to remove residual EPS. Because of the limiting amount of genomic DNA for AL-N samples, one paired-end Illumina library was prepared for AL-N-1 to 15. Three different libraries were prepared for the AL-W sample, which can be considered as some kind of technical triplicates. Two of them (AL-W-1a and AL-W-1b) were prepared from DNA fragments that had undergone a purification step to select for DNA fragments in the 150–250 bp size-range. AL-W-1a and AL-W-1b differed in the starting DNA amount used to construct the paired-end Illumina libraries (0.5 and 1 μg, respectively). The AL-W-1c paired-end Illumina library was constructed, as the four AL-N libraries, using a classical protocol with DNA fragments ranging from 100 to 500 bp in length. DNA from the seven metagenomic libraries was sequenced in two independent runs (AL-N and AL-W samples) using Illumina HiSeq2000 v3 (2x100 bp paired-end reads) by Beckman Coulter Genomics (Danver, MA, USA). The total number of paired end reads ranged from 78 to 131 millions, i.e., 7.8–13.1 Gbp per library and orientation (forward and reverse; **Table 2**).

**Table 2:** Sequence data obtained for the different Alchichica microbialite samples.

Series	AL-W (Western shore)			AL-N (Northern shore)			
Sample name	AL-W-1a	AL-W-1b	AL-W-1c	AL-N-1	AL-N-5	AL-N-10	AL-N-15
No. of paired-end reads^a^	78,303,604	97,861,012	130,622,648	130,153,392	110,282,579	97,849,068	131,562,915
No. of merged paired-end sequences	73,489,994	92,948,217	29,025,147	35,194,890	31,151,216	36,299,961	56,224,872
Average sequence length (bp)^b^	144	144	157	158	157	155	156
No. of total sequences affiliated to 16S + 18S rRNA genes^c^	22,713	24,697	13,533	15,276	13,694	14,819	35,038
Percentage of 16S + 18S rRNA genes	0.03	0.02	0.04	0.04	0.04	0.04	0.06
No. of bacterial 16S rRNA gene sequences (% singletons)	16,712 (14.6)	19,267 (11.5)	9,165 (10.5)	14,266 (7.0)	12,431 (5.4)	12,710 (5.6)	32,078 (5.0)
No. of chloroplast 16S rRNA gene sequences (% singletons)	3,617 (6.6)	2,652 (10.3)	2,253 (12.6)	251 (2.8)	293 (2.0)	729 (0.8)	1,076 (1.4)
No. of 18S rRNA gene sequences (% singletons)	2,374 (20.7)	2,766 (15.8)	2,113 (15.5)	717 (18.5)	927 (13.6)	1,374 (12.4)	1,876 (16.8)
No. of archaeal 16S rRNA gene sequences (% singletons)	10 (0.0)	12 (0.0)	2 (0.0)	42 (0.0)	43 (2.3)	6 (0.0)	8 (25.0)

*^a^Raw number of total pairs of reads produced (it implies the same number of forward and reverse sequences).*

*^b^Average length of assembled paired-end reads retained.*

*^c^Sequences longer than 130 bp only, including singletons.*

### Sequence Analyses

For each library, forward and reverse reads of a same DNA fragment (paired-end sequences) were assembled using the software FLASH (Magoc and Salzberg, 2011) with default parameters (overlap minimum, 10 nt; maximum allowed ratio between the number of mismatched base pairs and the overlap length, 0.25). Sequences corresponding to 16S/18S rRNA genes were searched using the nhmmer script implemented in the software HMMER, version 3.1b1 (Eddy, 2009). The 16S/18S rDNA HMM-profile matrices corresponding to the three domains of life used for the search were produced from a multiple alignment file available on SILVA website^1^, release 108. Although it has been shown that 16S rDNA amplicons as short as 100 bp are sufficient for accurate affiliation to high ranking taxonomic groups (Liu et al., 2007), we applied a more conservative criterion and included in our analysis only sequences longer than 130 bp to avoid potential problems derived from the fact that we were dealing with more complex, metagenomic datasets. Because the metagenomic 16S/18S rDNA sequences identified cover different regions of the 16S/18S rDNA gene, it was not possible to cluster our sequences in operational taxonomic units (OTUs). However, when sequences overlapped, identified 16S/18S rDNA sequences with 100% identity over their entire length were clustered with CD-HIT^2^ (Li and Godzik, 2006). This allowed us to group strictly identical sequences and use the longest sequence from each cluster to more accurately affiliate sequences from that cluster to a particular taxon using BLAST (Altschul et al., 1997). Representative cluster sequences and singletons (defined here as unique sequences, sequences that did not match any other sequence in our metagenomic datasets) were blasted against a reference database made of both 16S rDNAs (Silva SSU Ref NR 115; Quast et al., 2013) and 18S rDNAs (PR2 database; Guillou et al., 2013). A maximum *e*-value cut-off of 1*e*–5 was applied to affiliate sequences to particular taxa. We applied further filters at this step to eliminate sequences that might not correspond to actual rDNA fragments or that had only an rDNA portion (sequences at terminal end of genes and extending beyond). Sequences from a cluster were discarded if the coverage of their representative sequence with its best hit was lower than 95%. Similarly, singletons having a coverage lower than 95% and a sequence identity lower than 98% with their best hit were discarded. In general, because of the short length of the sequences retained (130–190 bp), taxonomic affiliation was considered reliable only above the Order level. The clustering process was only used to affiliate sequences to taxa; for quantitative analysis of relative abundances, we subsequently considered the number of sequences included in the clusters. Chloroplast sequences were considered independently. Statistics regarding the total number of reads and paired-end sequences per sample, the number of predicted 16S/18S rRNA genes and the average sequence lengths retained are presented in **Table 2**. The sequences analyzed in this work have been deposited in GenBank under the BioProject number PRJNA286185.

### Statistical Analysis

Statistical analyses were carried out using the R software^3^ (R Development Core Team, 2013). Non-metric multidimensional scaling (NMDS) ordination analyses were conducted on Bray–Curtis dissimilarities (Bray and Curtis, 1957) between community structure in the different microbialite samples, using the ‘Vegan’ R package (Oksanen et al., 2011) implemented in the R software^3^. Bray–Curtis distances corresponded to frequencies of sequences belonging to high-rank archaeal, bacterial, and eukaryotic taxa. No further transformation was applied to the data prior to the analysis. A Mantel test based on the Bray–Curtis distance matrix and a matrix of Euclidean distances of the physico-chemical parameters observed (mineral composition and depth) was applied to test whether microbial diversity was significantly correlated to the environmental variables. Mantel tests were done using the ‘Vegan’ package. Canonical Correspondence Analyses (CCA) including cyanobacterial order frequencies and mineral components were calculated with the ‘Ade4’ package.

## Results and Discussion

### Microbialite Samples and Identification of 16S/18S rRNA Gene Sequences in Metagenomic Datasets

We searched for 16S/18S rRNA gene sequences in seven metagenomic datasets produced in parallel from a total of five microbialite samples collected at two sites in the Northern and Western lake shores (**Figure 1**). The AL-N series corresponded to four microbialite fragments collected along a depth gradient from 1 to 15 m depth. We chose this site to be able to compare our high-throughput and more quantitative metagenome-based diversity results with those of a previous study based on PCR amplification, cloning, and Sanger sequencing analysis conducted in the same site and at similar depths (0.5 to 14 m; Couradeau et al., 2011). The AL-W sample was collected at 0.5–1 m depth at the Western shore. The site was chosen on the basis of often visible seepage activity (bubbling) and the different macroscopic morphology of microbialites. Two types of microbialites were present on this shore, brownish columnar microbialites and the cauliflower whitish hydromagnesite-dominated microbialites more common in the lake (Kazmierczak et al., 2011). Although the microbialite fragment that we collected at the AL-W site belonged to the latter, more conspicuous class of Alchichica microbialites and had a similar mineralogical composition to those in the AL-N site (**Table 1**), the microbial biofilm that covered its surface was less mineralized and had a more glutinous aspect (**Figure 1**). This resulted in a much higher DNA yield for AL-W, which allowed us to construct three independent genomic libraries for Illumina sequencing that we subsequently treated as technical replicates. Two of them, AL-W-1a and AL-W-1b, were constructed from size-fractionated DNA fragments comprised between 150 and 250 bp; AL-W-1c corresponded to a genomic library without DNA size fractionation.

Sequencing resulted in 78–131 million paired-end (forward and reverse) reads for each sample (**Table 2**). In the case of AL-W-1c and the AL-N series, between 22 and 42% of these reads could be merged in paired end sequences applying strict overlapping criteria (see Materials and Methods), which produced sequences of 150 bp on average. Unsurprisingly, libraries of size-selected DNA fragments (AL-W-1a and AL-W-1b) yielded much higher merging rates for paired end reads (∼95%). The merged paired-end datasets thus obtained for the different samples were mined for 16/18S rRNA genes belonging to the three domains of life. The proportion of predicted rDNAs was comparable among samples, ranging from 0.02 to 0.06%, which represented, despite the use of stringent quality filters, as many as 13,533–35,038 16S/18S rRNA gene sequences (**Table 2**).

**FIGURE 3:**
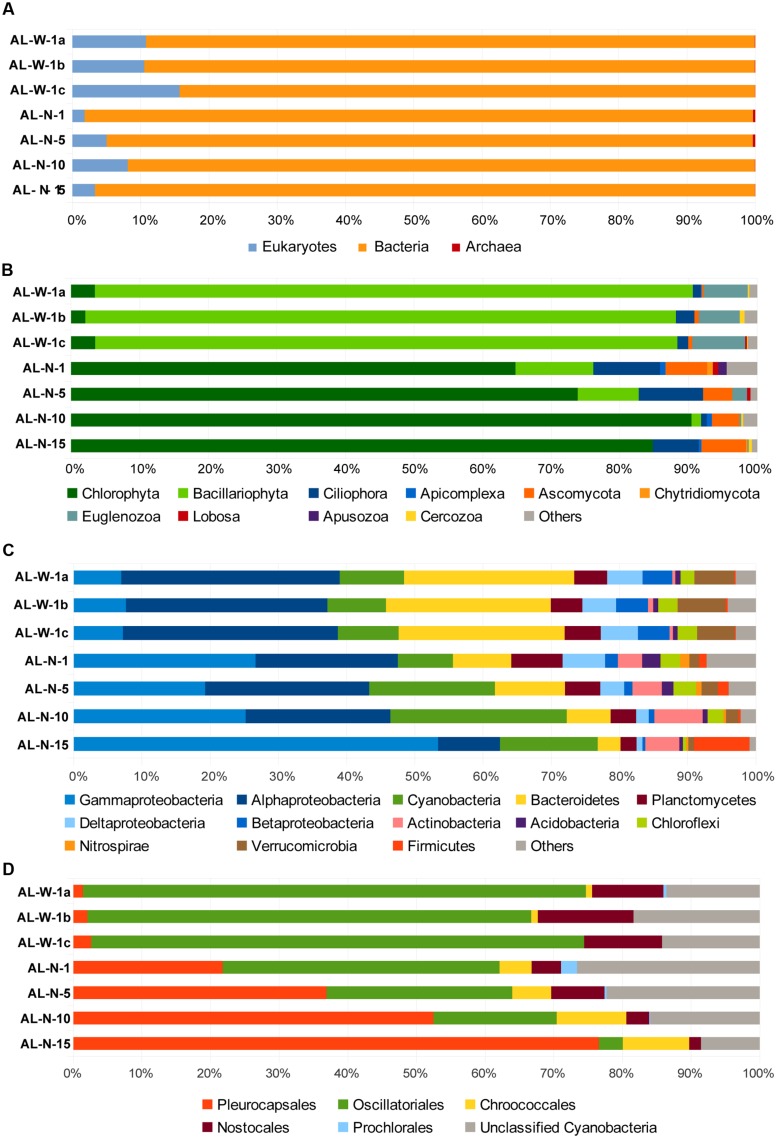
**Histograms showing the relative abundance of 16S/18S rRNA gene sequences in Alchichica microbialite metagenomes assigned to major different high-rank taxa. (A)** Proportion of sequences assigned to the three domains of life: archaea, bacteria and eukaryotes to the exclusions of metazoans (see also Supplementary Figure S1). **(B)** Phylogenetic clades of microbial eukaryotes, **(C)** bacterial phyla or classes (for the Proteobacteria), and **(D)** cyanobacterial orders. Neither singletons nor chloroplasts were included (their proportions are shown in Supplementary Figures S2–S4).

In order to get a more accurate taxonomic affiliation of the most abundant sequences in our datasets, we first clustered sequences that were 100% identical along their whole length (see Materials and Methods). We then chose the longest sequence as representative of the whole cluster and BLAST it against public databases to get a more accurate taxonomic affiliation of the cluster. However, for subsequent quantitative analysis, only the number of individual sequences was taken into account. Individual sequences that did not match any other sequence in each of our metagenomic datasets were analyzed independently. In the following, we refer to these unique sequences as ‘singletons’ by analogy with unique amplified sequences in amplicon studies. However, contrary to singletons in amplicon studies, a portion of which corresponds to sequencing artifacts or PCR chimeras, metagenomic singletons are unlikely to be artifacts and likely reflect lower abundances of the corresponding taxa. Metagenomic singletons accounted for ca. 5–15% of the predicted 16S/18S rRNA genes in our datasets (**Table 2**). Comparing the phylogenetic affiliation and relative abundance of clustered sequences and singletons should reveal if patterns of phylogenetic diversity differed significantly between high and low abundant organisms. However, the distribution of relative proportions of singletons and of more abundant sequences among major bacterial and eukaryotic taxa were remarkably similar (compare **Figure 3** and Supplementary Figure S2). This suggests, with minor exceptions (see below), that low-abundant organisms essentially correspond to low-abundance species or genera within the same dominant high-rank taxa (orders or phyla), and not to the occurrence of novel or rare divergent taxa.

### General Patterns of Microbial Diversity Across Samples

Two key advantages result from direct, PCR-free, metagenomic-based diversity analyses. First, it is possible to access to 16S/18S rRNA genes for all organismal diversity simultaneously, including archaea, bacteria, and eukaryotes. Second, because PCR-induced quantitative differences are avoided, the number of rDNA sequences identified in metagenomes reflects the corresponding relative abundance of genes present in the environment. However, although copy number may vary across taxa, especially in the case of eukaryotes, and might not reflect relative cell abundance, quantitative PCR and mock community analyses suggest that rDNA copy number may be a good proxy for biomass (Godhe et al., 2008; Egge et al., 2013). Of course, cell lysis and DNA purification biases might exist, which we tried to minimize by pooling DNA from multiple DNA purification experiments (20–32 for each sample). In the case of Alchichica microbialites, the overall distribution of 16S/18S rRNA genes in the three domains of life confirmed previous ideas suggesting that bacteria dominate this kind of samples, with roughly 85–95% sequences, whereas archaea were excessively minor components (<1%), consistently with other studies (Goh et al., 2009; Couradeau et al., 2011). Eukaryotes varied from 2–8% in the AL-N samples to 10–15% in the AL-W samples (**Figure 3A**). These values are comparable to those found in metagenomic datasets from Cuatro Ciénegas (around 10% eukaryotic sequences; Breitbart et al., 2009) or the Bahamas (less than 13%; Khodadad and Foster, 2012).

We subsequently analyzed the diversity of 16S/18S rRNA gene sequences at a finer level of taxonomic resolution within the bacteria and the eukaryotic domains. Archaea encompassed only a few sequences in all the metagenomes (**Table 2**) and will not be commented further. Both Thaumarchaeota and Euryarchaeota sequences were detected in all samples, except AL-N-10 and AL-N-15 where only Thaumarchaeota were present (data not shown). This corroborated the presence of the two archaeal lineages previously detected (Couradeau et al., 2011). Most reads matched poorly characterized sequences, although at least some Euryarchaeota belonged to the Haloarchaea. Whether these archaea are truly associated to the microbialites or are planktonic archaea from the surrounding waters remains to be determined. By contrast, bacterial and eukaryotic sequences were much more abundant and diverse. The within-domain bacterial and eukaryotic diversity patterns observed for the three AL-W replicate samples were remarkably similar (**Figure 3**; Supplementary Figures S1 and S2). This strongly suggests that the patterns observed for the depth profile (AL-N samples) are also reliable even if replicates could not be obtained for these samples due to the much lower DNA yield.

Although we tried to eliminate little invertebrates feeding on the biofilm, larvae or eggs when collecting our samples, a significant portion of metazoan sequences were detected, ranging from almost 10 to 60% of the total 18S rDNAs depending on the metagenome (Supplementary Figure S1). These essentially affiliated to Arthropoda (mainly Acari), Annelids, and Nematoda. Even if some metazoans are truly living in close association with the microbialites within the more superficial, less mineralized zones of the biofilm, e.g., nematodes, and might play a control on the microbial community (Tarhan et al., 2013), many of the identified sequences corresponded to arthropods or other exogenous animals that skewed the relative abundance of the various taxa toward metazoan members. Therefore, we subsequently excluded metazoan sequences from our datasets to compare the relative distribution of the rest of eukaryotic taxa (**Figure 3B**). The eukaryotic diversity covered a large variety of high-rank taxa in all the samples, but was numerically dominated by photosynthetic taxa that contributed between 75 and 95% of the microbial eukaryotic sequences. However, whereas AL-W metagenomes were dominated by diatom sequences (almost 90% of microbial eukaryotic sequences), samples from the AL-N vertical profile were all dominated by green algal sequences (**Figure 3B**). This radical difference between the two sites does not appear related to the mineral composition of the substrate (**Table 1**) but may be related to the local hydrochemistry, which seems locally influenced by seepage activity and perhaps also by occasional spring discharge at the steepest part of the crater. Indeed, the chemical analysis of seepage water collected at this site in 2007 revealed much less saline (668 mg/l of total dissolved solids versus ca. 8,800 mg/l for the lake water) but far more Si-rich water. Si was on average 40 times more concentrated (694 μmol/l) than the rest of the water lake (Kazmierczak et al., 2011). This local Si input is likely determinant for the dominance of diatoms in these samples. The differences in the eukaryotic component of the surface biofilm may partly explain the distinct macroscopic aspect of the biofilm (**Figure 1**). In addition to diatoms and green algae, likely photosynthetic euglenids (Euglenozoa, Excavata) were present in the two sites, having a noticeable percentage (6–8%) in the AL-W site. The heterotrophic component of the microbial eukaryotic community was more varied and encompassed protist grazers (ciliates, amoebae, cercozoans, or apusozoans) and fungi. Some apicomplexan sequences were identified, which might correspond to parasites (**Figure 3B**). The analysis of singletons revealed the presence of some groups not detected in high abundance; for instance, haptophytes were identified in AL-N-1, AL-N-10, and AL-N-15 (Supplementary Figure S2B).

Metagenome sequences were largely dominated by bacteria, which were also highly diverse, encompassing numerous phyla (**Figure 3C**). Although in varying proportions, Cyanobacteria, Gammaproteobacteria and Alphaproteobacteria, and Bacteroidetes were generally the most abundant groups, collectively accounting for up to 70 or 80% of all bacterial sequences, depending on the metagenome. Gammaproteobacteria were nevertheless much less abundant in AL-W samples (<10% bacterial sequences) as compared to the AL-N samples, where they accounted for 20% up to more than 50%. Bacteroidetes, on the contrary, were more abundant in the AL-W samples, accounting for 20% of sequences. Cyanobacteria were less abundant in surface microbialites (around 10%), but increased their proportions to 15–30% in deeper samples. Other relatively abundant groups (5–10% sequences) comprised the Planctomycetes, Deltaproteobacteria, Chloroflexi, Verrucomicrobia and, in the AL-N samples, also the Gram positive Actinobacteria and, in the deeper sample AL-N-15, Firmicutes. Many other phyla and candidate divisions were detected in minor amounts. The phylogenetic distribution of singletons was consistent with that of more abundant sequences (Supplementary Figure S2).

Because cyanobacteria are thought to play an essential role in microbialite formation (Dupraz et al., 2009; Obst et al., 2009) and because we had detected a shift of cyanobacterial diversity with depth in a previous study based on amplicon cloning and sequencing (Couradeau et al., 2011), we studied in more detail the relative abundance of the main cyanobacterial orders identified with confidence (**Figure 3D**). AL-W samples were largely dominated by filamentous cyanobacteria of the order Oscillatoriales, followed by Nostocales. However, in the AL-N samples, although AL-N-1 was also dominated by Oscillatoriales, their proportion clearly diminished with depth. On the contrary, members of the Pleurocapsales were much more abundant in the 15 m deep sample, shifting from 20% to almost 80% of the cyanobacterial 16S rRNA gene sequences identified. Therefore, our more quantitative metagenome-based analyses clearly confirm the trend previously observed (Couradeau et al., 2011).

### Microbial Taxa Potentially Promoting Carbonate Precipitation in Microbialites

Photosynthesis and sulfate reduction are thought to be the most important microbial metabolisms promoting carbonate precipitation (Dupraz and Visscher, 2005). Unlike other energy metabolisms, photosynthesis and sulfate reduction can be phylogenetically targeted to specific microbial groups. Accordingly, the relative contribution of different microbial taxa to carbonate precipitation can be in principle assessed from the relative proportion in metagenomes of 16S/18S rRNA gene sequences corresponding to taxa carrying out those types of metabolism. Although rRNA gene copy number varies across taxa, it has been shown that it varies proportionally to cell biomass in photosynthetic eukaryotes (Godhe et al., 2008; Egge et al., 2013), and it is also proportional to cell complexity, itself related to cell size, in cyanobacteria (Schirrmeister et al., 2012). We can therefore hypothesize that the relative abundance of rRNA genes in Alchichica metagenomes reflects the relative biomass of the different taxa they affiliate to and, potentially, their respective impact on carbonate formation. Based on this premise, we extracted from the different metagenomes the 16S/18S rRNA gene sequences corresponding to taxa with typical photosynthetic or sulfate reducing metabolisms, which we classed in four different categories: cyanobacteria, photosynthetic eukaryotes, anoxygenic phototrophs, and sulfate reducers. In the case of AL-W, since the three replicate samples were highly congruent, we combined the sequences from the three replicates. Both cyanobacteria and photosynthetic eukaryotes, containing cyanobacteria-derived chloroplasts, carry out oxygenic photosynthesis, whereas a variety of bacteriochlorophyll-containing bacterial lineages can perform anoxygenic photosynthesis, including Chlorobi, many Chloroflexi, and several Alphaproteobacteria (e.g., *Rhodospirillum, Rhodobacter*) and Gammaproteobacteria (e.g., *Chromatium, Ectothiorhodospira*) (Bryant and Frigaard, 2006). Similarly, members of several deltaproteobacterial orders (e.g., Desulfobacterales, Desulfuromonadales, Desulfovibrionales) and some Firmicutes genera (*Desulfotomaculum*, *Desulfosporomusa*, and *Desulfosporosinus*) (Madigan et al., 2014) are sulfate reducers. Because sequences are short (130–190 bp), depending on the level of sequence conservation of the region of the 16S/18S rRNA gene that they cover, it may be difficult to ascribe them to a particular genus or even family. We only retained sequences with clear BLAST hits to those genera, families and orders. This implies that the relative abundances that we recovered correspond to minimal estimates for the different groups, and most especially for lower-rank taxa (diverse anoxygenic photosynthetic and sulfate-reducing genera and families).

**FIGURE 4:**
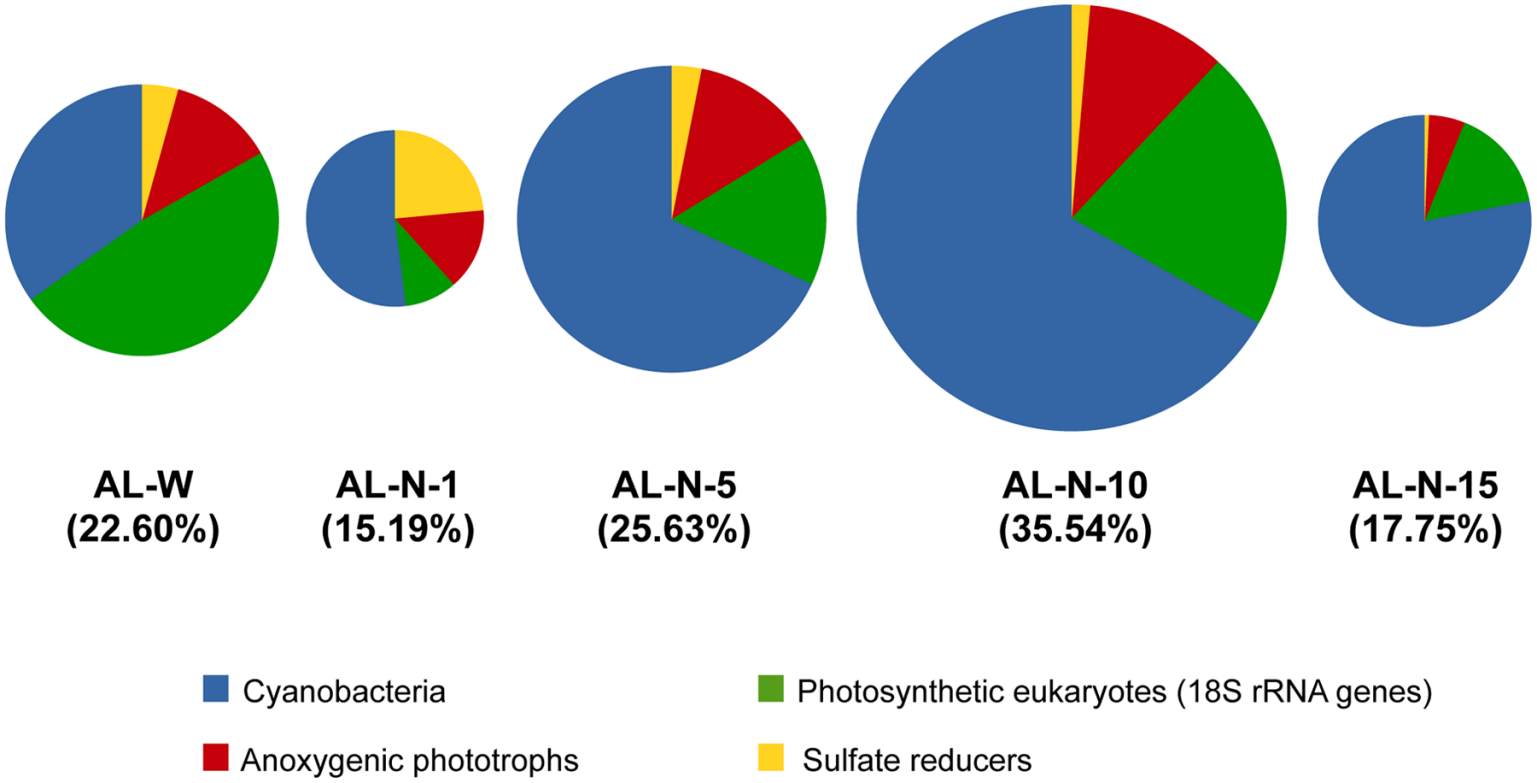
**Relative abundance of 16S/18S rRNA gene sequences affiliated to lineages displaying metabolisms favoring carbonate precipitation in Alchichica microbialites.** Sizes of pie charts (and the values within brackets) indicate the total proportion represented by these sequences compared to the total number of rRNA gene sequences in each sample. Chloroplasts are not included (to see the proportion of photosynthetic eukaryotes based on chloroplast sequences see Supplementary Figure S4).

Between 15 and 35% of the total number of 16S/18S rRNA gene sequences in each metagenome corresponded to lineages potentially promoting carbonate precipitation, most of them corresponding to oxygenic or anoxygenic photosynthesizers (**Figure 4**). This implies that the remaining rRNA gene sequences (between ca. 65 and 85% of the total) corresponded to non-photosynthetic organisms, most of which are likely heterotrophs carrying out aerobic or anaerobic respiration or fermentation. Their metabolic activity would tend to counterbalance the carbonate-promoting action of photosynthesis and sulfate reduction (Dupraz and Visscher, 2005). Consequently, for net carbonate precipitation to occur, photosynthesis and/or sulfate reduction must be more active than respiration and/or the hydrochemical conditions override the acidification resulting from respiration and fermentation. Interestingly, although the proportion of cyanobacterial sequences was abundant and even dominated in AL-N samples, between roughly 25% and up to almost 70% of the carbonate biomineralization potential corresponded to others groups. Notably, although the total relative proportion of all eukaryotic rDNA sequences represented from 2 to 15% at most (**Figure 3A**), photosynthetic eukaryotes accounted for higher proportions among the organisms with carbonate precipitation potential, reaching up values of 20 to 50% in some samples (respectively, AL-N-10 and especially the diatom-dominated AL-W metagenome; **Figure 4**). Although anoxygenic photosynthesizers varied, they reached similar relatively high (∼15%) proportions in AL-W and all AL-N samples but AL-N-15, where they were less abundant. Finally, sulfate reduction, although relatively minor in all the microbialite fragments analyzed, was relatively abundant in AL-N-1, ca. 20%. The higher proportion of 16S rRNA genes affiliated to anoxygenic phototrophs and sulfate reducers in this sample might in principle suggest that the sampled microbialite fragment (or the mineral grains that we detached from all over the surface for the analysis, see Materials and Methods) contained larger portion of anoxic zones than the other microbialite fragments. However, there might be alternative or additional explanations not related to global anoxia, since typically anaerobic sulfate reducers have been conspicuously observed to be abundant and very active in oxic areas of microbial mats (e.g., Teske et al., 1998; Minz et al., 1999). Also, anoxygenic phototrophs can establish symbioses within sheaths of filamentous cyanobacteria belonging to the Oscillatoriales (D’Amelio et al., 1987). Coincidentally, members of the Oscillatoriales are the dominant cyanobacteria in the AL-N-1 microbialite fragment. Finally, it might also be that the collected fragment (or part of it) was placed in a relatively shadowed area, and this particular situation might have selected for anoxygenic phototrophs, which are adapted to lower light intensities. At any rate, this observation indicates that the microbial communities vary within small spatial scales in response to the heterogeneous local physico-chemical conditions. It also suggests that anoxygenic photosynthesis and sulfate-reduction can be responsible for an important fraction of carbonate precipitation in relatively oxygen-poor microbialite zones, making them potentially interesting areas to study as analogs of stromatolites formed in the Precambrian before oxygen levels significantly raised (Bosak et al., 2007; Farquhar et al., 2011).

Nonetheless, oxygenic photosynthesis seems prevalent in modern Alchichica microbialites. Overall two different kinds of patterns were observed, with eukaryotic photosynthesis dominating AL-W and cyanobacterial photosynthesis dominating AL-N samples. Around 50% of the 18S rRNA genes belonging to taxa with carbonate-induction potential corresponded to photosynthetic eukaryotes, essentially diatoms, in AL-W. In this sample, the number of chloroplast 16S rRNAs as compared with the nuclear 18S rRNA genes of photosynthetic eukaryotes was higher, suggesting more chloroplasts per cell than in the AL-N samples (Supplementary Figure S3). Therefore, if the number of chloroplast genes is considered the eukaryotic contribution in AL-W samples appears even higher (Supplementary Figure S4). However, in this particular sample, given the local hydrochemistry influenced by the input of less saline and neutral pH waters (Kazmierczak et al., 2011) and the less mineralized nature of the biofilm growing on the microbialite surface, the overall biomineralization potential of this specific community is difficult to assess and might be questioned. It might be envisaged that the diatom fraction grows to the expense of Si-rich fresh seepage waters and that carbonate precipitation occurs at very local scale, deeper in the biofilm, promoted by a subset of the microorganisms more intimately associated with the microbialite and/or at some periods of the year when seeping activity is less important. The fact that Si concentration in the AL-W microbialite is comparable, and even smaller, than in the AL-N microbialites favors this hypothesis (**Table 1**). This suggests that the potential diatom input to the microbialite silicon content (**Table 1**; Zeyen et al., under reveiw) is not different from that of the rest of microbialites in the lake or, alternatively, that Si in the microbialites has not a diatom origin. On the contrary, the AL-N samples were strongly mineralized with many cyanobacteria and green algae intimately linked with the mineral substrate. This suggests that at least part of this diversity may be directly involved in biomineralization.

### Effect of Depth and Chemical Parameters on Microbial Community Structure and Macroscale Evidence for Aragonite Biomineralization by Pleurocapsales

**FIGURE 5:**
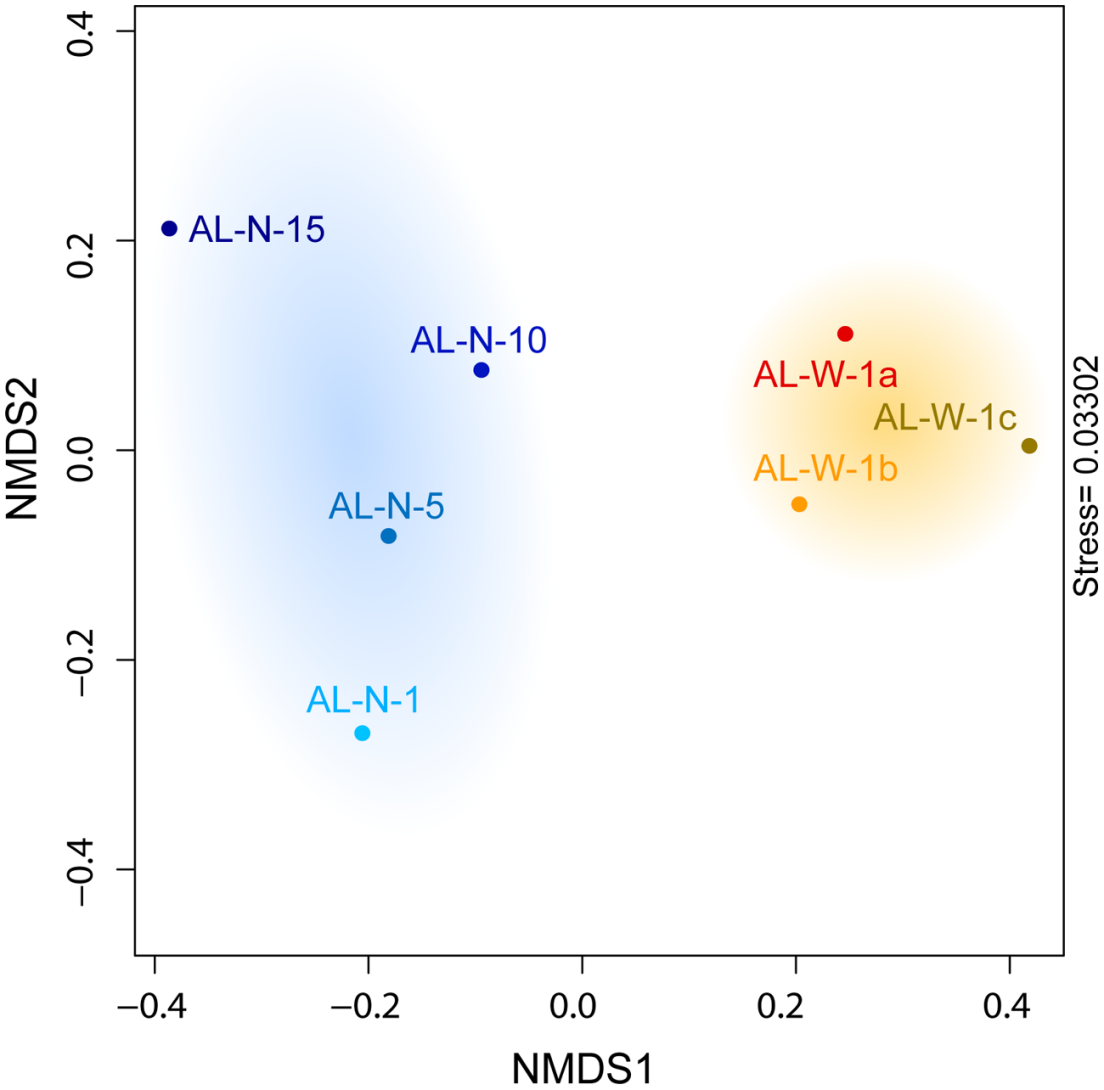
**Non-Metric Multi-dimensional Scaling (NMDS) plot showing differences in microbial community composition between all seven samples for both eukaryotes and prokaryotes**.

We looked for potential correlations between the community composition and the local physico-chemical parameters measured, essentially depth and the chemical composition of the microbialite fragments that we analyzed in parallel (**Table 1**). First, ordination analyses of bacterial plus eukaryotic community composition at the level of major taxa (**Figures 3B,C**) using NMDS clearly separated the different samples according to their location and depth (**Figure 5**). As expected, the three AL-W replicate samples clustered together, and were segregated from the AL-N samples along the NMDS1 axis. AL-N samples were discriminated along the second axis (NMDS2) according to depth. Similar results were observed when eukaryotes and prokaryotes were analyzed separately (data not shown).

We then used Mantel tests to investigate the significance of the correlations linking environmental parameters to the observed microbial communities. We found no significant correlation between the overall microbial diversity (eukaryotes and prokaryotes, all samples considered) and the chemical composition of the microbialites (*r* = 0.35, *p-value* = 0.24). Remarkably, Mantel tests revealed a significant correlation between depth and overall microbial community composition (*r* = 0.577, *p-value* = 0.035). Even if the number of points is limited at this stage, these results indicate that at least depth is an important determinant of community structure. However, it is likely that microbial community structure depends also on other local abiotic parameters. For instance, the influence of seepage water on local hydrochemistry (Kazmierczak et al., 2011) may explain the diatom dominance on AL-W microbialites. In addition, biotic interactions, although more difficult to assess in such complex communities, likely play an important role.

**FIGURE 6:**
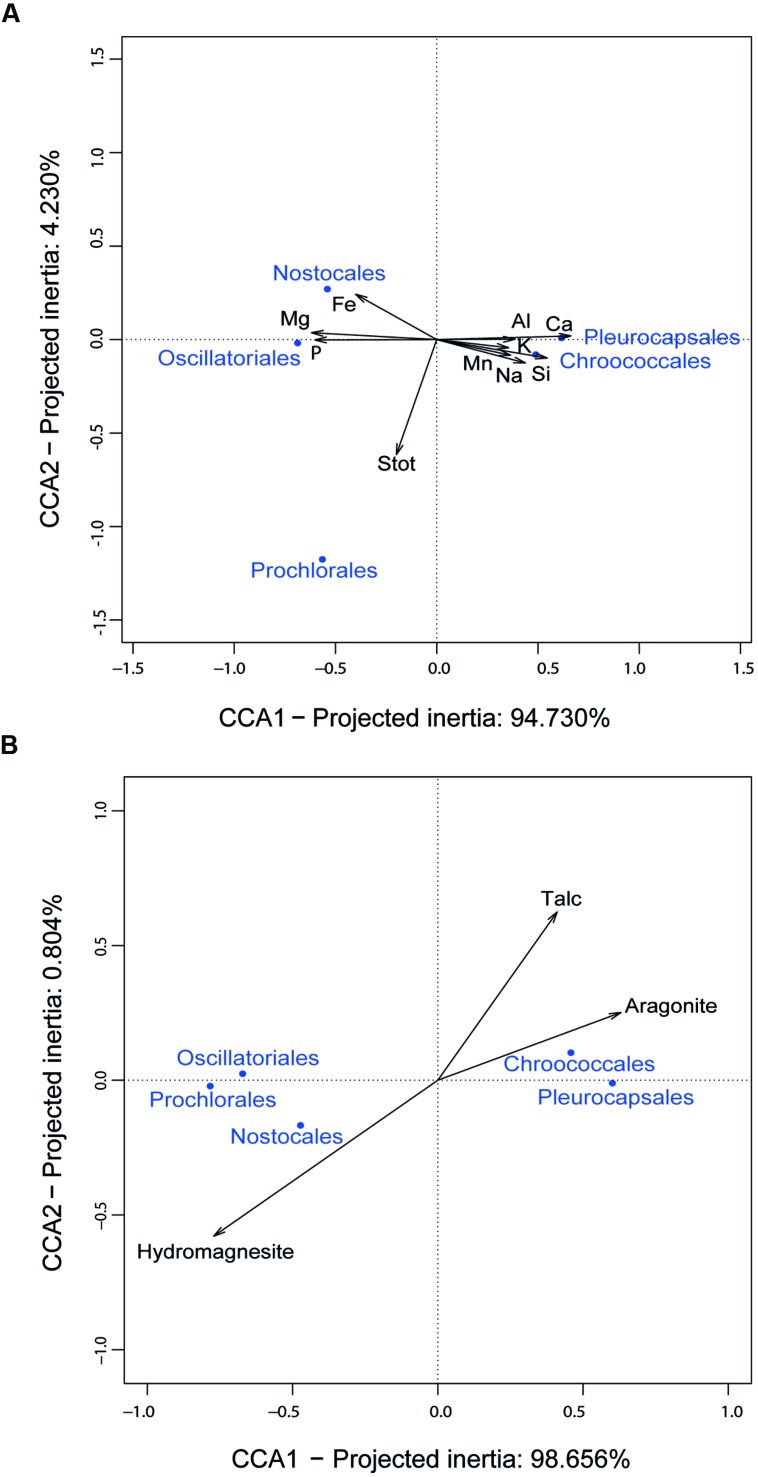
**Canonical Correspondence Analysis (CCA) of 16S rRNA gene sequence frequencies of dominant cyanobacterial taxa and chemical parameters of Alchichica microbialites. (A)** CCA including the elemental composition of microbialites; **(B)** CCA including the mineral composition of microbialites.

The fact that Mantel tests did not show a significant correlation between the overall community structure and the mineral composition of the rock might be the consequence of the high diversity and complexity of the microbial community. However, the situation might be different for taxa specifically involved in biomineralization. Since cyanobacteria generally appeared the most important contributors to the carbonate precipitation potential (40–80% depending on the samples; **Figure 4**), we looked for potential correlations between the chemical and mineral composition of microbialites and the cyanobacterial taxa identified. CCA revealed relationships between some cyanobacterial orders and particular chemical parameters. Pleurocapsales and Chroococcales strongly and positively correlated with the calcium content of microbialites (**Figure 6A**) and, therefore, with the only mineral phase carrying Ca in these microbialites, i.e., aragonite (**Figure 6B**). On the opposite trend, the Nostocales correlated with magnesium and its associated mineral in modern Alchichica microbialites, hydromagnesite (**Figure 6**). Prochlorales and Oscillatoriales did not show any particular correlation with the chemical/mineralogical composition. The latter is not surprising. Prochlorales are typically small planktonic cyanobacteria, particularly well studied in oceans (Biller et al., 2015). Their presence in Alchichica microbialites was minor (**Figure 3D**), suggesting a contamination from the surrounding lake plankton. Oscillatoriales, on the contrary, were the dominant cyanobacteria in surface samples from the AL-W and AL-N sites although their global presence is limited since, in those samples, cyanobacterial genes accounted for less than 10% of the total bacterial 16S rRNA genes (**Figure 3C**). Oscillatoriales are typical filamentous cyanobacteria that live on the surface of the microbialites and often depend on high light intensities (van der Grinten et al., 2005). They use them as support but are not embedded within the substrate (Couradeau et al., 2011, 2013; Gerard et al., 2013), which might explain the absence of correlation observed. Nostocales correlated with the presence of hydromagnesite, which is the dominant carbonate in Alchichica microbialites. Many *Nostoc*-related heterocyst-containing cyanobacteria are endolithic (Sigler et al., 2003). Nostocales-like cyanobacteria have been observed in Alchichica microbialites well embedded in the carbonate-associated biofilms (Kazmierczak et al., 2011; Gerard et al., 2013). A positive correlation with hydromagnesite in this context might simply reflect the fact that these cyanobacteria get embedded in the carbonate mineral that massively constitutes the microbialites. Whether they actively or passively favor magnesium carbonate formation, eventually getting trapped in the mineral, or whether they eventually induce its local dissolution by an active dissolution process as has been described for some endolithic cyanobacteria (Garcia-Pichel et al., 2001), which would also result in the specific correlation with hydromagnesite, remains an open question.

The positive correlation of Pleurocapsales and Chroococcales with calcium and aragonite was particularly interesting. Importantly, the variance explained by the axis separating these two orders and aragonite/talc from the rest represented more than 98% (**Figure 6**), indicating a very strong correlation and arguing in favor of causality. Previous studies at microscale have shown a specific connection between members of the Pleurocapsales and aragonite using a variety of microscopy and spectroscopy techniques (Couradeau et al., 2013; Gerard et al., 2013). Confirming previous observations, SEM imaging of thin sections of the microbialite fragments analyzed in this study show extensive areas (several hundreds of microns) occupied by aragonite encrusted *Pleurocapsa*-like cyanobacteria (**Figures 2D,E**). Our CCA analysis based on metagenome-inferred diversity and the mineral composition of microbialites show that same trend at macroscale, reinforcing the idea that cyanobacteria belonging to the Pleurocapsales are indeed responsible for the biomineralization of aragonite in Alchichica microbialites. Interestingly, Chroococcales showed a similar positive correlation with aragonite. Since members of the Chroococcales are coccoid cyanobacteria endowed with thick mucilaginous sheaths, properties shared with the Pleurocapsales, and are often associated with microbialites and microbial mats in extreme environments (Komárek and Anagnostidis, 1999), it is tempting to hypothesize that they may be also responsible for the specific biomineralization of aragonite. This might perhaps be due to a similar capability of chelating calcium ions in their EPS sheaths and making them available for carbonate precipitation upon local EPS degradation by heterotrophic bacteria. Exploring the specific connection between Chroococcales and aragonite formation at microscale will be necessary to test this idea.

At any rate, given the high relative abundance of Pleurocapsales (and perhaps also, though to a lesser extent, Chroococcales), which induce aragonite formation extracellularly before being filled by aragonite post-mortem (Couradeau et al., 2013), it seems clear that extracellular carbonate precipitation is the dominant biomineralization mechanism acting in the formation of Alchichica microbialites. This is so despite the fact that two cyanobacterial lineages able to form intracellular carbonates, represented by *Gloeomargarita lithophora* and *Synechococcus calcipolaris*, were detected and enriched from Alchichica microbialites (Couradeau et al., 2012; Benzerara et al., 2014; Ragon et al., 2014). However, confirming a previous study suggesting that they were not abundant (Couradeau et al., 2011) and despite intensive sequencing effort, we were unable to identify 16S rRNA gene sequences related to these two lineages in the analyzed microbialite fragments. This indicates that, while present, these lineages are in very low abundance and do not contribute significantly to present carbonate formation in these systems. This does not necessarily imply that their ancestors, which likely lived in different geochemical environments, were not able to significantly contribute to carbonate formation, something that might potentially explain the enigmatic absence of cyanobacterial microfossils in Archaean stromatolites (Couradeau et al., 2012; Riding, 2012).

## Conclusion

In this manuscript, we make a series of observations about the diversity of different taxa associated to living microbialites based on metagenome sequence data and try to infer potential players in carbonate precipitation. Function cannot be directly inferred from metagenomic sequence data without making a series of assumptions that are reasonable (based on known diagnostic metabolisms for specific lineages) but that may be affected by many different biases that are common to all molecular- and metagenomic-based studies (microbial activity vs. dormancy, DNA extraction biases, sequencing biases). This cautionary note implies that our metagenome-based diversity data allow postulating functional hypotheses that need to be validated by complementary approaches. In our case, by mining metagenomes generated by direct high-throughput Illumina sequencing of microbialites from the Alchichica lake collected at two distinct sites and at different depths in search of 16S and 18S rRNA gene sequences, we have been able to establish a more quantitative and deeper estimation of the microbial diversity associated to these structures. Our results confirm and extend previous observations based on classical gene amplification, cloning, and sequencing (Couradeau et al., 2011), which revealed very similar trends in terms of large major taxa. Our study reveals that, despite the overwhelming dominance of bacteria, most of them correspond to likely heterotrophic lineages, whereas only a fraction (roughly 15–30% total rRNA gene sequences) correspond to lineages with potential carbonate precipitation-promoting metabolic activities. Even if cyanobacteria account for the most important contribution to those, sulfate reducing bacteria (Deltaproteobacteria and some Firmicutes) and, especially, photosynthetic anoxygenic lineages (essentially some Gammaproteobacteria and Alphaproteobacteria genera and some Chloroflexi) represent significant fractions of potential contributors to carbonate precipitation. Compared with bacteria, although eukaryotic 18S rRNA gene sequences constituted only 2–15% of all sequences, most eukaryotic sequences corresponded to photosynthetic eukaryotes, whereas archaea were anecdotic components in these microbialites. Despite some common trends in the relative presence of the most abundant taxa, we observed clear differences in the microbial community composition between the two studied sites and at different depths. The eukaryotic diversity of the sample analyzed in triplicate for the Western shore was dominated by diatoms, in detriment of cyanobacteria, and had different proportions of major bacterial taxa. This community structure was likely influenced by the local Si-rich freshwater seepage observed (Kazmierczak et al., 2011). Microbialites collected in the Northern shore at different depths (1–15 m depth) exhibited more mineralized biofilms and, accordingly, their microbial diversity resembled more, as compared to that of the Western shore. In the Northern shore samples, green algae were the dominant photosynthetic eukaryotes, although photosynthetic bacteria, either cyanobacteria or anoxygenic photosynthesizers, and sometimes sulfate reducers were more relatively abundant. Microbial community composition varied with depth. The most important trend was a shift in cyanobacterial composition, which was dominated by Oscillatoriales in the surface and by Pleurocapsales in deeper samples, where cyanobacteria were more abundant. Despite the required caution in interpreting sequence data, in the specific case of Pleurocapsales, we find a strong support for their key role as promoters of aragonite precipitation extracellularly. This includes (i) published SEM, TEM, CLSM, and STXM analyses at micrometer scale of progressively aragonite-encrusted *Pleurocapsa*-like cells (Couradeau et al., 2013; Gerard et al., 2013), (ii) observation of pavement-like areas of encrusted Pleurocapsales covering hundreds of micrometers (this work) and (iii) statistical correlation of Pleurocapsales abundance and aragonite in otherwise hydromagnesite-dominated microbialites (this work). Future studies (e.g., on the ability of *Pleurocapsa* sp. retrieved from these microbialites to induce aragonite precipitation in culture) will allow testing whether our hypothesis of a causal link holds true. In addition, we also hypothesize that Chroococcales, less abundant than Pleurocapsales but following similar trends, may be involved in aragonite biomineralization. By contrast, despite the previous detection of intracellularly calcifying cyanobacteria in Alchichica microbialites, their proportion in our metagenome-based diversity data is negligible, suggesting that extracellular precipitation is the major source of biomineralization in these structures.

## Conflict of Interest Statement

The authors declare that the research was conducted in the absence of any commercial or financial relationships that could be construed as a potential conflict of interest.

## Footnotes

1 http://www.arb-silva.de

2 http://weizhongli-lab.org/cd-hit/

3 http://cran.r-project.org
