# Supplementary material for: Metagenome-based diversity analyses suggest a significant contribution of non-cyanobacterial lineages to carbonate precipitation in modern microbialites
Source: Front Microbiol. 2015 Aug 5;6:797. doi: 10.3389/fmicb.2015.00797 (PMC4525015; doi:10.3389/fmicb.2015.00797)
Supplement: Supplementary file 1 [file Presentation_1.PDF]

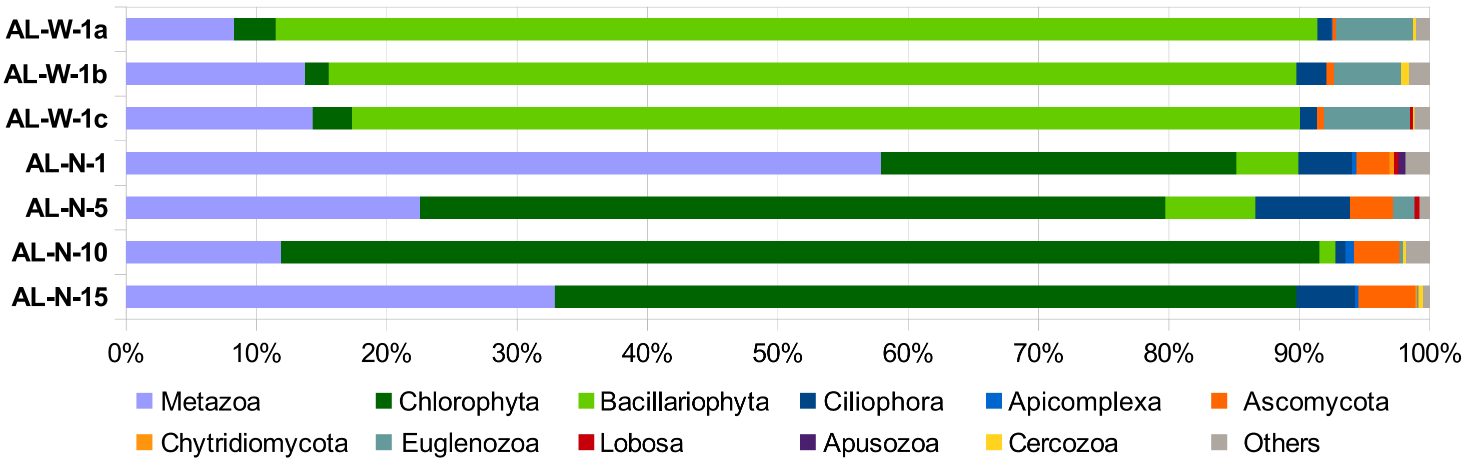


**Figure S1.** Histogram displaying the relative proportion of all 18S rRNA gene sequences, including Metazoans detected in AL-N and AL-W microbialite metagenomes.

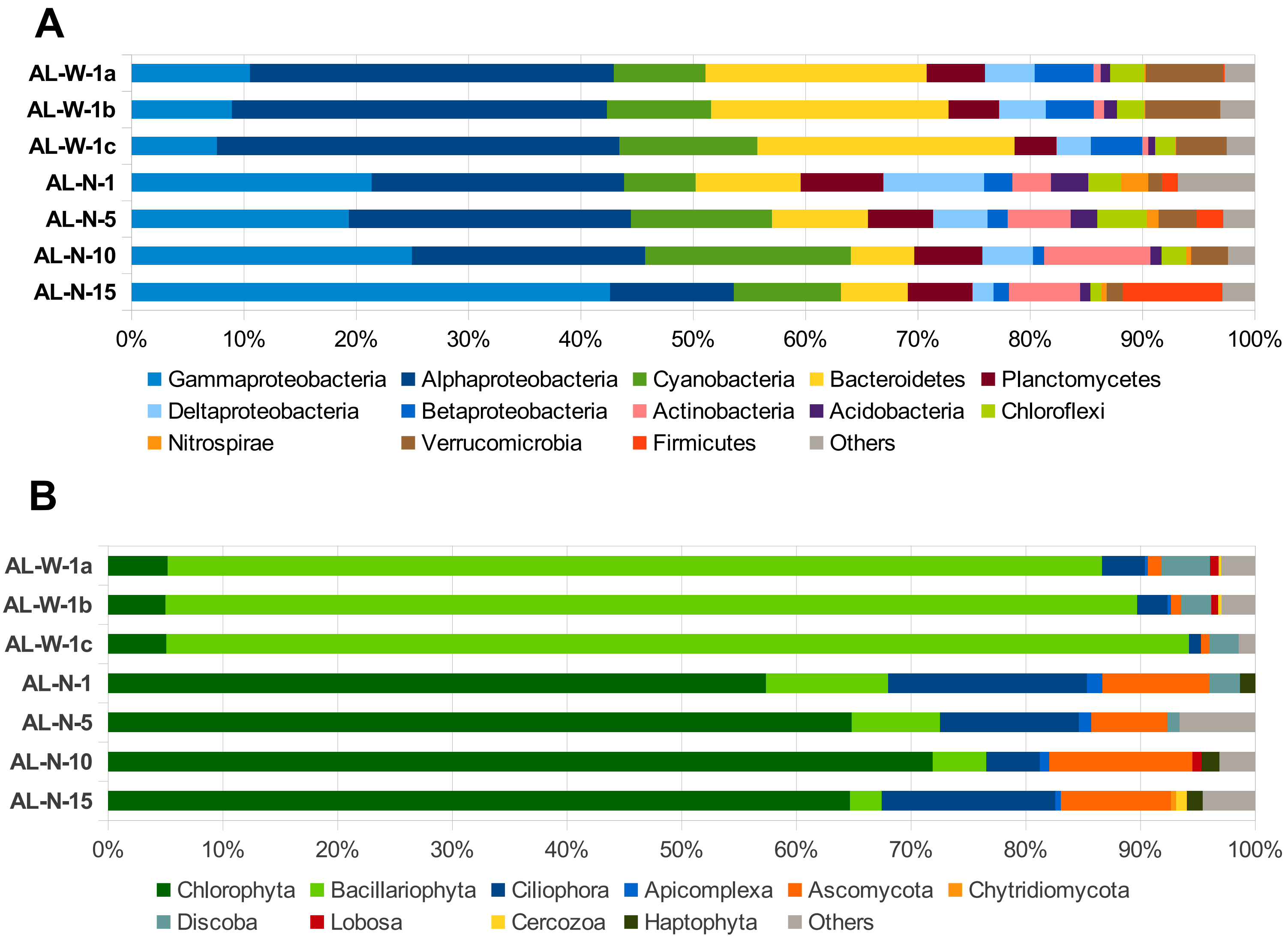


**Figure S2.** Histogram displaying the relative proportion of singletons assigned to (A) bacterial 16S rRNA genes and (B) 18S rRNA genes in AL-N and AL-W microbialite metagenomic datasets (Metazoa excluded).

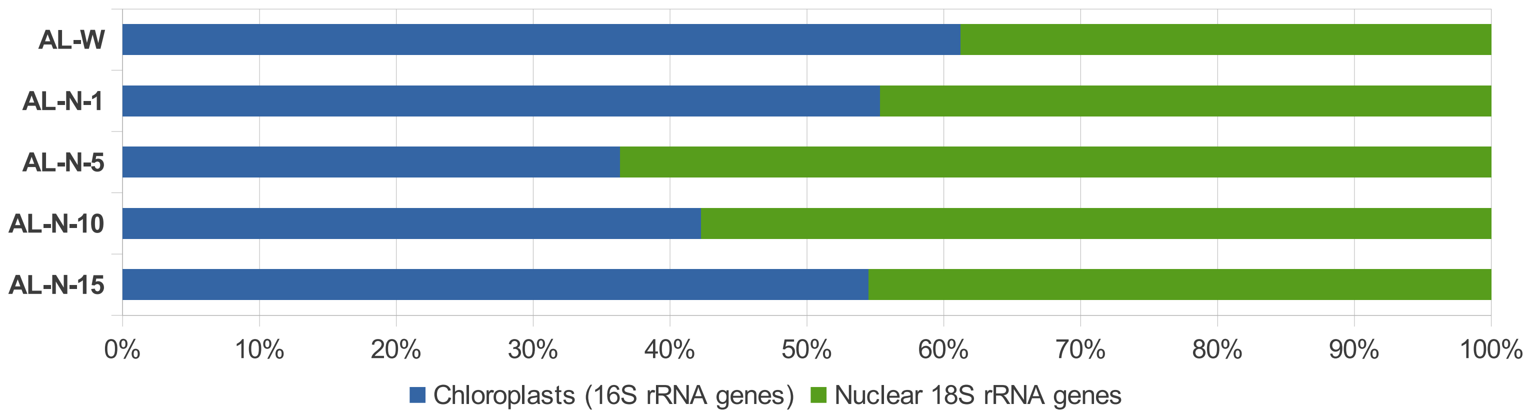


**Figure S3.** Relative proportion of chloroplast 16S rRNA and nuclear 18S rRNA genes detected in the different Alchichica microbialite metagenomes. The three AL-W replicates are merged in a single histogram bar.

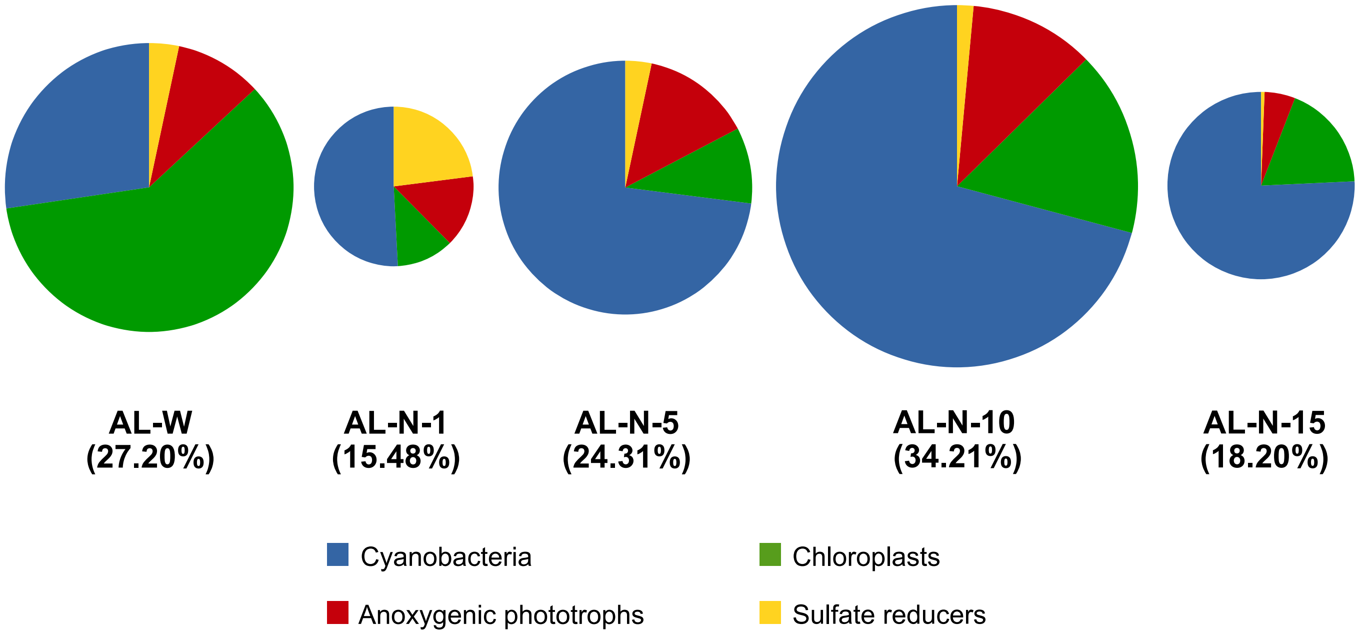


**Figure S4.** Relative abundance of 16S rRNA gene reads affiliated to lineages displaying metabolisms favoring carbonate precipitation in Alchichica microbialites. 16S rRNA chloroplast genes are used to represent photosynthetic eukaryotes. Sizes of pie charts (and the values within brackets) indicate the total proportion represented by these sequences compared to the total number of rRNA gene sequences in each sample.
